# Asystole in a young child with tetrahydrocannabinol overdose: a case report and review of literature

**DOI:** 10.3389/ftox.2024.1371651

**Published:** 2024-05-09

**Authors:** Mats Steffi Jennifer Masilamani, Rebecca Leff, Yu Kawai

**Affiliations:** ^1^ Division of Pediatric Cardiology, Department of Pediatrics and Adolescent Medicine, Mayo Clinic, Rochester, MN, United States; ^2^ Department of Emergency Medicine, Mayo Clinic, Rochester, MN, United States; ^3^ Division of Pediatric Critical Care Medicine, Department of Pediatrics and Adolescent Medicine, Mayo Clinic, Rochester, MN, United States

**Keywords:** children, edibles, THC overdose, asystole, Case Report

## Abstract

**Introduction:**

The association between Δ8-tetrahydrocannabinol (THC) and cardiac dysrhythmia has not been well described in children. Asystole, while consistent with reports of severe bradycardia and apnea in children, is uncommonly described in the current literature. We present the first pediatric case of asystole and apnea following THC ingestion.

**Case:**

A 7-year-old male presented to the emergency department (ED) after his mother noticed he was lethargic 3–4 h after accidental ingestion of five 15 mg (total of 75 mg) Δ8-THC gummies. Upon arrival, he was vitally stable and well-appearing. He received maintenance intravenous fluids. Approximately 7 h after initial ingestion, he experienced a >15-s episode of asystole and apnea on telemetry requiring sternal rub to awaken. This was followed by bradycardia (60 beats per minute range) which resolved with 0.1 mg glycopyrrolate. He was admitted to the PICU, drowsy but arousable with stable vitals. After an uneventful 24-h (post-ingestion) PICU observation, he was discharged home in stable condition.

**Discussion:**

To our knowledge, this is the first reported pediatric case of THC-induced asystole. The etiology of asystole may be attributed to direct vagal stimulation of THC or respiratory depression. The typical recommended observation time after potential toxicity is 3–6 h after children have returned to their physiological and behavioral baseline. Our patient was clinically stable with no concern for respiratory depression or cardiac dysrhythmia yet experienced an asystolic pause with apnea 7 h after initial ingestion.

**Conclusion:**

Our case demonstrates that asystole and apnea may occur in pediatric patients following large THC ingestions and those symptoms can appear late outside of the currently recommended observation period.

## Introduction

Over the past 5 years, increased edible cannabis exposures in children have resulted in increased hospital visits and admission to pediatric intensive care units (PICUs) in the United States (Leonard et al., 2022). Reviewing the National Poison Data System (NPDS) from 2000 to 2020, it was identified that the proportion of children in the registry with unintentional cannabis ingestion admitted to the PICU increased from 9.5% in 2000 to 14% in 2020 (Leonard et al., 2022).

Δ9-tetrahydrocannabinol (THC) was the most readily available THC; now due to the perceived legal loophole in the Agriculture Improvement Act of 2018 (formerly known as Farm Bill), Δ8-THC is more widely available, being added in high concentrations to edible products particularly in attractive labels (United States H.R, 2018). A recent study indicated that the amount of Δ8-THC available in the products sold in retail stores had amounts that can cause intoxication (Kaczor et al., 2024).

Unlike adult patients with large Δ9-THC exposures who typically present with tachycardia, anxiety, paranoia, and hallucinations, pediatric patients with significant THC exposure may present with significant central nervous system (CNS) depression, ataxia, respiratory depression, apnea, bradycardia, hypotension, and seizures (Shaker et al., 2023; Akpunonu et al., 2021; Burrows and Williams, 2019; Wong and Baum, 2019; Wang et al., 2016; Wang et al., 2014). Increasing reports of bradycardia and apnea are particularly concerning as the cardiovascular effects of THC are not fully understood (Idris et al., 2022).

Asystole, while consistent with reports of severe bradycardia and apnea in children, is uncommonly described in the current literature (Brancheau et al., 2016). Further understanding of the mechanism of severe bradycardia and asystole in THC toxicity in children is paramount, considering that the wide range of THC edibles that are currently available have palatable appeal and resemble candy.

The typical recommended observation time after potential toxicity is 3–6 hours (h) with or without telemetry once children have returned to prior health status (Camarena-Michel and Zuckerman, 2024; Uptodate, 2024). Here, we present a case of a seven-year-old child who had a 15 seconds (s) episode of asystole and apnea that occurred 7 hours after initial THC edible ingestion, requiring medical intervention and PICU admission.

## Case presentation

**FIGURE 1:**
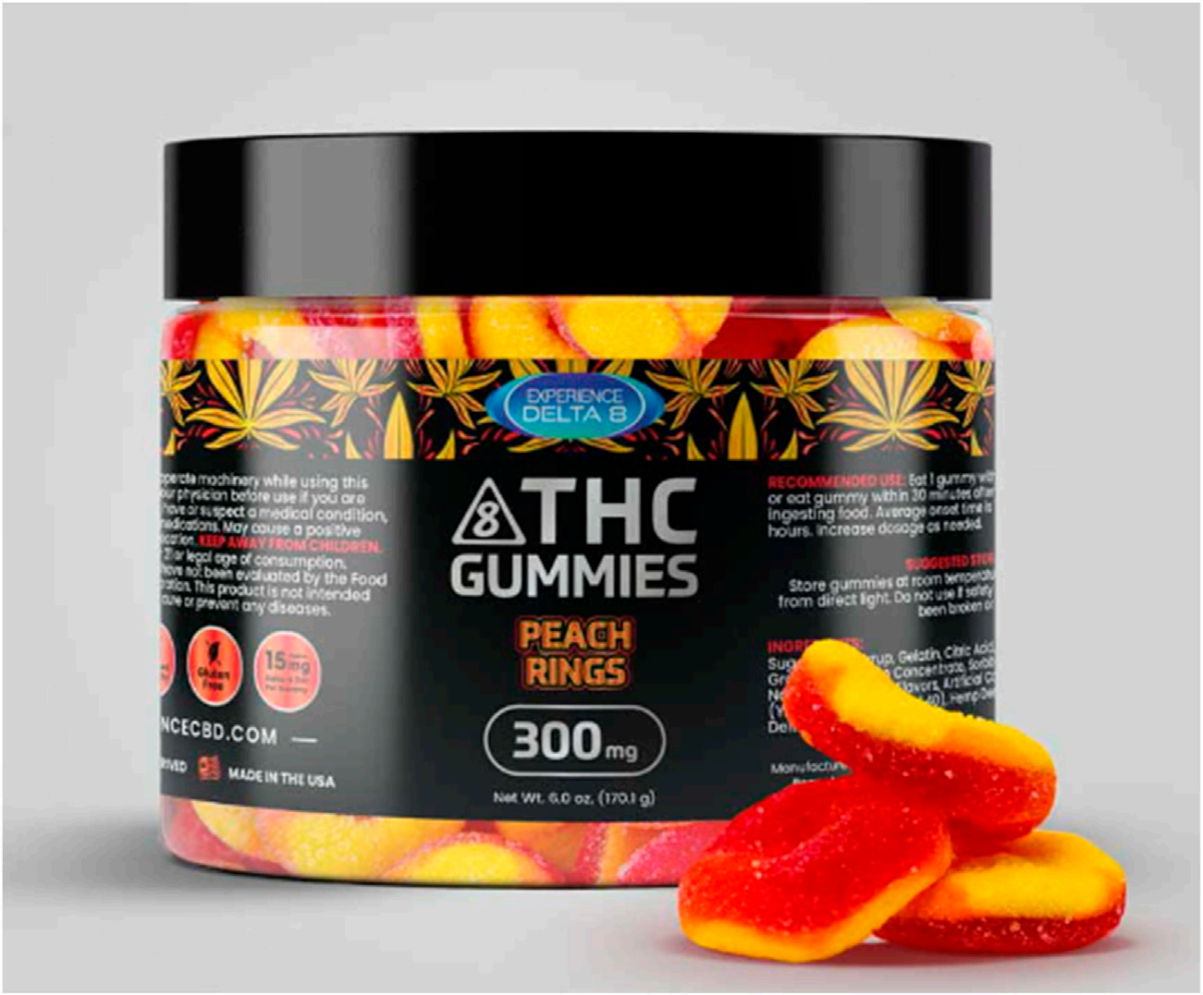
Δ8-tetrahydrocannabinol edible gummies.

A 7-year-old male (25 kg) with a history of Attention-Deficit and Hyperactivity Disorder (ADHD) presented to an outside hospital emergency department (ED) around 8:00 p.m. after his mother noticed he was lethargic 3–4 h after accidental ingestion of five 15 mg (total of 75 mg) Δ8-THC edible gummies (Figure 1). Of note, there were no other accessible medicines or illicit drugs in the house. He was on guanfacine 1 mg daily for ADHD. His vital signs on presentation to the ED were unremarkable: heart rate 91 beats per minute (bpm), blood pressure 85/50 mmHg, respiratory rate 13 breaths per minute, and oxygen saturation of 99% on room air. Baseline labs were obtained: comprehensive metabolic panel was notable only for glucose of 181 mg/dL (normal: 70–99 mg/dL). Magnesium level was 1.9 mg/dL (normal 1.7–2.1 mg/dL). Creatine Kinase was not elevated, 77 U/L (normal: 20–200 U/L). Alcohol, aspirin, and acetaminophen levels were undetectable. Electrocardiogram (EKG) showed normal sinus rhythm with incomplete right bundle-branch block, normal intervals (QTC 420 ms), and no significant ST-segment elevation or depression. Incomplete right bundle-branch block is not an uncommon finding in a healthy school-aged child (Meziab et al., 2018).

**FIGURE 2:**
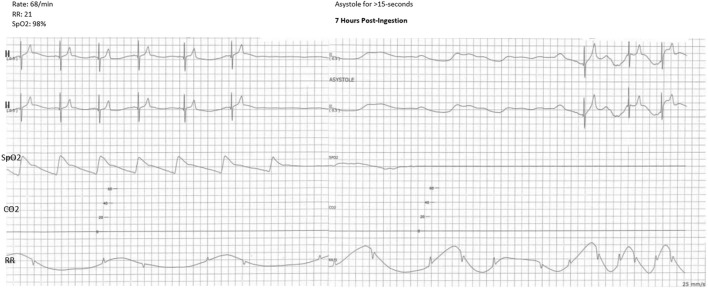
Telemetry strip showing asystole for >15-s.

He received maintenance IV fluids but no additional intervention. Around 11:00 p.m., approximately 7 hours after initial ingestion, the patient remained sleeping but was easily arousable with no additional signs of toxicity. When awakened, he was alert and oriented. He ambulated without difficulty to urinate then returned back to bed and fell asleep. About 5 minutes after that event, the telemetry alarm went off, indicating >15-s episode of asystole and apnea (Figure 2). The patient did not awaken to regular stimulation and required a sternal rub that led to return of respiration and increase in heart rate. No associated seizure activity was observed. After the episode, the patient returned back to sleep. His heart rate was noted to be bradycardic in the 60-bpm range, so he received one dose of glycopyrrolate 0.1 mg, which resolved the bradycardia. He was then transferred to our PICU for further monitoring.

When he arrived to the PICU, he was alert and oriented, although slightly slow in his thoughts. Vital signs were stable: heart rate 72 bpm, blood pressure 120/69 mmHg, normal respiratory effort, and oxygen saturation of 99% on room air. The physical exam was unremarkable with no focal neurological deficits and no evidence of respiratory depression or apnea. He continued to progress well clinically and hemodynamically with no recurrence of asystole. After an uneventful period of observation in the PICU, he was discharged home approximately 24 h after ingestion with recommendation to follow up with his primary care physician in 2 days and sooner if there were any changes in his physiological and/or behavioral baseline. He did well at follow up and family had no concerns.

Given the clear history, no quantitative THC blood tests were performed. His urine drug screen (UDS) obtained 24 h after ingestion was positive for THC and negative for other illicit substances.

## Discussion

The association of THC and cardiac dysrhythmia has not been well described in a young child. To our knowledge, this is the first reported case of THC-induced asystole in a child.

### Pharmacology

**FIGURE 3:**
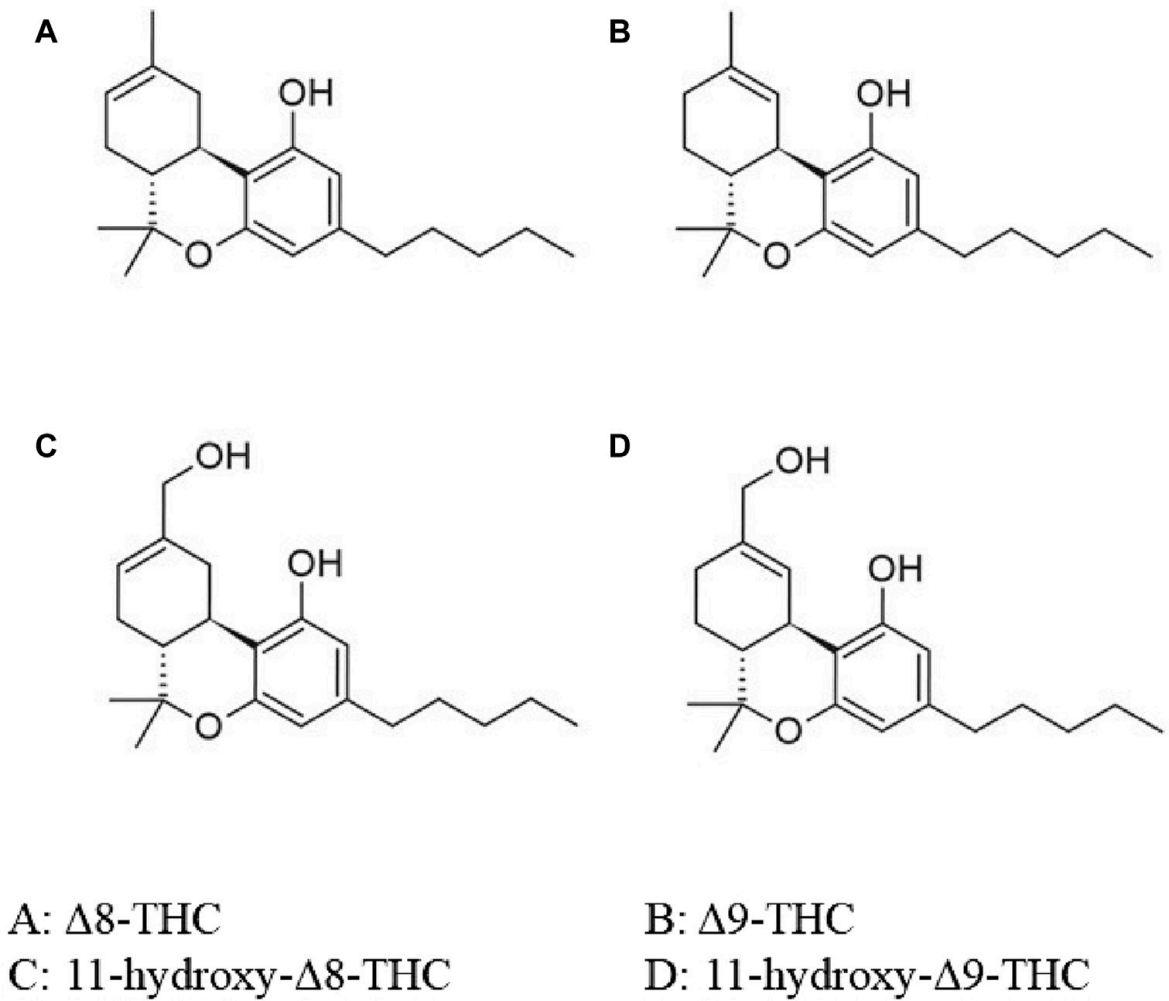
Chemical structure of Δ8-THC, Δ9-THC and its metabolites. O, oxygen; OH, hydroxide; THC, tetrahydrocannabinol. Adapted from Tagen and Klumpers (2022)

The active ingredient in marijuana or cannabinoid (CB), is known as THC. The most commonly referenced and typically, the most plentiful substance is the psychoactive cannabinoid, Δ9-THC. Δ8-THC is a structural isomer of Δ9-THC (Figure 3) (Tagen and Klumpers, 2022). Δ9-THC is nearly completely metabolized by hepatic hydroxylation and oxidation through cytochrome P450 (CYP 450) isozymes to an active and more potent primary metabolite, 11-hydroxy-Δ9-THC (11-OH-Δ9-THC). Similarly, Δ8-THC is metabolized to a more potent 11-hydroxy-Δ8-THC (11-OH-Δ8-THC) via the same metabolic pathway (Figure 3) (Tagen and Klumpers, 2022).

THC works on two major receptors: CB1 and CB2 receptors. CB1 receptors are neuronal receptors present in the central nervous system, and CB2 receptors are expressed in immune cells. CB1 and CB2 have been demonstrated in myocardium and vascular endothelium. An earlier study indicated that Δ9-THC and Δ8-THC have similar binding affinities to CB1 and CB2 receptors (Babalonis et al., 2021). However, a more recent study suggested that Δ8-THC binds to CB1 with less affinity than Δ9-THC but has a similar binding affinity to CB2 (Tagen and Klumpers, 2022). Existing reports suggest that Δ9-THC and Δ8-THC oral products produce similar effects, and Δ8-THC is thought to be generally less potent than Δ9-THC (Hollister and Gillespie, 1973). However, the potency difference between the 11-OH metabolites may not be equivalent to the potency difference between the parent Δ9-THC and Δ8-THC. Given that large quantities of Δ8-THC are chemically synthesized, little is known about the comparative clinical aspect of the now available Δ8-THC to that of Δ9-THC (Tagen and Klumpers, 2022).

### Cardiovascular effects

Although CB1 and CB2 receptors have been demonstrated in myocardium and vascular endothelium (Mukhopadhyay et al., 2007), because of the abundance of those receptors in the brain, cardiac manifestations of THC are considered to be through its activity on the autonomic nervous system. Cardiovascular effects of THC are biphasic: at low or moderate doses, there is a surge in sympathetic activity causing tachycardia and hypertension, and at higher doses, there is prominent parasympathetic stimulation resulting in bradycardia and hypotension (Fisher et al., 2005). A variety of cardiovascular effects of THC has been described in adults, from dysrhythmias including atrial tachyarrhythmias (atrial tachycardia, flutter, and fibrillation), ventricular tachyarrhythmias (ventricular tachycardia and fibrillation), symptomatic bradyarrhythmia, complete atrioventricular block, vasovagal syncope, sudden cardiac death due to atherosclerosis in the coronary arteries, and coronary vasospasm in normal coronaries causing ischemia and sudden cardiac death (Richards et al., 2020; Mathew et al., 2003; Kasuda et al., 2021). Brancheau et al. reported a 28-year-old male who had THC-induced asystole (Brancheau et al., 2016).

The etiology of asystole, in this case, could possibly be attributed to profound direct vagal stimulation and/or THC-induced CNS depression and somnolence causing apnea that then led to a strong autonomic system modulation producing extreme bradycardia and asystole (Koehler et al., 2011). Extreme vagal stimulation prolongs the atrial refractory period, causing bradycardia.

### Current recommendations

American Association of Poison Control Centers (AAPCC) recommends healthcare facility referral for all symptomatic children (drowsiness, mental status change, persistent vomiting), children who are expected to sleep within 3 h of exposure, and asymptomatic and symptomatic children who are exposed to amounts greater than their weight-based threshold for age (6 months-5 years: >0.2 mg/kg; 6–12 years: >0.3 mg/kg; 13–79 years: >0.4 mg/kg) (Supplementary Material S1). Our patient ingested 75 mg of THC, or, 3 mg/kg, which is 10 times the weight-based threshold for his age. Half-life of THC in plasma is about 30 h (Ashton, 2001). Inhaled cannabis has a rapid onset of action with peak effect in 15–30 min and lasts up to 2–3 h. Ingested cannabis has a delayed onset of action ranging from 30–90 min, peak effect in 2–3 h, and lasts up to 12 h (Grotenhermen, 2003).

Typically described toxic effects of THC in children are CNS-related (Shaker et al., 2023; Akpunonu et al., 2021). It has been shown that lethargy and somnolence along with the increased duration of clinical effect and length of hospital stay were most commonly observed in the THC-naïve group than the non-THC-naïve group (Richards et al., 2020). Toxicity in children usually occurs after ingestion of a highly concentrated product. The current edible THC products in the form of gummies, and suckers are obviously attractive to children as they resemble candy, and this could lead to ingestion in large quantities resulting in intoxication. Continued free availability of such products call for some serious policy change consideration in order to protect the vulnerable population such as our patient.

The current recommendation is monitoring for 3–6 h (AAPCC: 3–4 h, UpToDate: 4–6 h) for asymptomatic children (Supplementary Material S1). Our patient was clinically stable with no concern for respiratory depression and had asystole and apnea after 7 h of ingestion. This is genuinely concerning as the life-threatening event occurred outside of the recommended observation time. In 2016, Heizer et al. did a retrospective chart review of children who had unintentional exposure to THC where they found the degree of symptoms corresponded to the amount of THC ingested and that correlated with the level of management they needed. As described in this paper, observation vs. hospital admission vs. ICU monitoring should be determined based on a weight-based threshold for age rather than how well they appear clinically at initial presentation (Heizer et al., 2018). We suggest it would be prudent to transfer children who ingest large amounts of THC to higher centers with ICU facilities.

## Conclusion

Our case demonstrates that asystole and apnea can occur following THC ingestion in children, and those symptoms can appear late outside of the currently recommended observation period. The unique symptomology could be explained by the significantly large ingestion of THC, amounting to 10 times his weight-based threshold to institute a medical observation.

### Patient perspective

“I was concerned when my son ingested the THC gummies, but I had no idea that while spending time at the Emergency Department, his heart would stop beating! I was not aware that something terrible could happen from having too much THC. My son received great care at the hospital, and I am so glad that he was discharged back to his usual self. I think the community should be aware that something like this could happen to a child. Thank you for allowing me to voice my thoughts!”

### Limitation

Although the THC substance consumed was a gummy with less chance of contamination with other illicit drugs, a comprehensive serum drug screen was not obtained.

## Data Availability

The original contributions presented in the study are included in the article/Supplementary Material, further inquiries can be directed to the corresponding author.

## References

[B1] Akpunonu P. Baum R. A. Reckers A. Davidson B. Ellison R. Riley M. (2021). Sedation and acute encephalopathy in a pediatric patient following ingestion of delta-8-tetrahydrocannabinol gummies. Am. J. Case Rep. 22, e933488. 10.12659/AJCR.933488 34762615 PMC8594112

[B2] Ashton C. H. (2001). Pharmacology and effects of cannabis: a brief review. Br. J. Psychiatry 178, 101–106. 10.1192/bjp.178.2.101 11157422

[B3] Babalonis S. Raup-Konsavage W. M. Akpunonu P. D. Balla A. Vrana K. E. (2021). Δ8-THC: legal status, widespread availability, and safety concerns. Cannabis Cannabinoid Res. 6 (5), 362–365. 10.1089/can.2021.0097 34662224 PMC8664123

[B4] Brancheau D. Blanco J. Gholkar G. Patel B. Machado C. (2016). Cannabis induced asystole. J. Electrocardiol. 49 (1), 15–17. 10.1016/j.jelectrocard.2015.10.003 26520167

[B5] Burrows K. Williams J. A. (2019). THC intoxication in a 16-month-old child. Paediatr. Child. Health 24 (5), 299–300. 10.1093/pch/pxz015 31379428 PMC6656951

[B6] Camarena-Michel A. Zuckerman M. (2024). Cannabis toxicity and poisoning. Available at: https://www.emrap.org/corependium/chapter/rec1Woa3JM0g9hPwq/Cannabis-Toxicity-and-Poisoning#h.mhab9ovl1fy9 .

[B7] Fisher B. A. C. Ghuran A. Vadamalai V. Antonios T. F. (2005). Cardiovascular complications induced by cannabis smoking: a case report and review of the literature. Emerg. Med. J. 22 (9), 679–680. 10.1136/emj.2004.014969 16113206 PMC1726916

[B8] Grotenhermen F. (2003). Pharmacokinetics and pharmacodynamics of cannabinoids. Clin. Pharmacokinet. 42 (4), 327–360. 10.2165/00003088-200342040-00003 12648025

[B9] Heizer J. W. Borgelt L. M. Bashqoy F. Wang G. S. Reiter P. D. (2018). Marijuana misadventures in children: exploration of a dose-response relationship and summary of clinical effects and outcomes. Pediatr. Emerg. Care 34 (7), 457–462. 10.1097/PEC.0000000000000770 27050740

[B10] Hollister L. E. Gillespie H. K. (1973). Delta-8- and delta-9-tetrahydrocannabinol comparison in man by oral and intravenous administration. Clin. Pharmacol. Ther. 14 (3), 353–357. 10.1002/cpt1973143353 4698563

[B11] Idris I. Diez J. R. Assoku B. A. Beker S. (2022). Accidental ingestion of tetrahydrocannabinol-laced gummies causing bradycardia and first-degree atrioventricular block in a pediatric patient: a case report. Cureus 14 (7), e26826. 10.7759/cureus.26826 35847165 PMC9278991

[B12] Kaczor E. E. Greene K. Babu K. M. Berthold E. C. Sharma A. Carreiro S. P. (2024). Commercial delta-8 THC products: an analysis of content and labeling. J. Med. Toxicol. 20 (1), 31–38. 10.1007/s13181-023-00974-y 37917314 PMC10774316

[B13] Kasuda S. Kondo T. Terazawa I. Morimoto M. Yuui K. Kudo R. (2021). Cardiac sudden death in a young cannabis user. Leg. Med. (Tokyo) 53, 101955. 10.1016/j.legalmed.2021.101955 34438239

[B14] Koehler U. Reinke C. Sibai E. Hildebrandt O. Sohrabi K. Dette F. (2011). Autonomic dysfunction and cardiac arrhythmia in patients with obstructive and central sleep apnea. Dtsch. Med. Wochenschr 136 (50), 2622–2628. 10.1055/s-0031-1292852 22160956

[B15] Leonard J. B. Laudone T. Hines E. Q. Klein-Schwartz W. (2022). Critical care interventions in children aged 6 months to 12 years admitted to the pediatric intensive care unit after unintentional cannabis exposures. Clin. Toxicol. (Phila) 60 (8), 960–965. 10.1080/15563650.2022.2059497 35384771

[B16] Mathew R. J. Wilson W. H. Davis R. (2003). Postural syncope after marijuana: a transcranial Doppler study of the hemodynamics. Pharmacol. Biochem. Behav. 75 (2), 309–318. 10.1016/s0091-3057(03)00086-8 12873621

[B17] Meziab O. Abrams D. J. Alexander M. E. Bevilacqua L. Bezzerides V. Mah D. Y. (2018). Utility of incomplete right bundle branch block as an isolated ECG finding in children undergoing initial cardiac evaluation. Congenit. Heart Dis. 13 (3), 419–427. 10.1111/chd.12589 29431296

[B18] Mukhopadhyay P. Bátkai S. Rajesh M. Czifra N. Harvey-White J. Haskó G. (2007). Pharmacological inhibition of CB1 cannabinoid receptor protects against doxorubicin-induced cardiotoxicity. J. Am. Coll. Cardiol. 50 (6), 528–536. 10.1016/j.jacc.2007.03.057 17678736 PMC2239316

[B19] Richards J. R. Blohm E. Toles K. A. Jarman A. F. Ely D. F. Elder J. W. (2020). The association of cannabis use and cardiac dysrhythmias: a systematic review. Clin. Toxicol. (Phila) 58 (9), 861–869. 10.1080/15563650.2020.1743847 32267189

[B20] Shaker K. Nillas A. Ellison R. Martin K. Trecki J. Gerona R. (2023). Delta-8-Tetrahydrocannabinol exposure and confirmation in four pediatric patients. J. Med. Toxicol. 19 (2), 190–195. 10.1007/s13181-022-00927-x 36757578 PMC10050257

[B21] Tagen M. Klumpers L. E. (2022). Review of delta-8-tetrahydrocannabinol (Δ8 -THC): comparative pharmacology with Δ9 -THC. Br. J. Pharmacol. 179 (15), 3915–3933. 10.1111/bph.15865 35523678

[B22] United States H.R (2018). “Agriculture improvement Act of 2018,” in 115th United States congress. United States H.R.2, Available at: https://www.congress.gov/bill/115th-congress/house-bill/2 .

[B23] Uptodate (2024). Cannabis (marijuana): acute intoxication - UpToDate. Available at: https://www.uptodate.com/contents/cannabis-marijuana-acute-intoxication .

[B24] Wang G. S. Le Lait M.-C. Deakyne S. J. Bronstein A. C. Bajaj L. Roosevelt G. (2016). Unintentional pediatric exposures to marijuana in Colorado, 2009-2015. JAMA Pediatr. 170 (9), e160971. 10.1001/jamapediatrics.2016.0971 27454910

[B25] Wang G. S. Roosevelt G. Le Lait M. C. Martinez E. M. Bucher-Bartelson B. Bronstein A. C. (2014). Association of unintentional pediatric exposures with decriminalization of marijuana in the United States. Ann. Emerg. Med. 63 (6), 684–689. 10.1016/j.annemergmed.2014.01.017 24507243

[B26] Wong K. U. Baum C. R. (2019). Acute cannabis toxicity. Pediatr. Emerg. Care 35 (11), 799–804. 10.1097/PEC.0000000000001970 31688799

