# Supplementary material for: Asystole in a young child with tetrahydrocannabinol overdose: a case report and review of literature
Source: Front Toxicol. 2024 May 9;6:1371651. doi: 10.3389/ftox.2024.1371651 (PMC11112079; doi:10.3389/ftox.2024.1371651)
Supplement: Supplementary file 1 [file DataSheet1.PDF]

Minnesota Poison Control System Treatment Guideline:

Cannabis (marijuana)

Pharmacology & Summary in Overdose/Poisoning:

Pharmacology

Cannabis is the most commonly consumed drug in the world,[1] and is generally well-tolerated.[2] As of 2022, it has also been approved for medicinal use by qualifying patients in 39 states and the District of Columbia, and is available for adult recreational use in 19 states. Though there are hundreds of chemical components in marijuana (including cannabinoids, terpenes, lipids, flavonoids, etc), the most commonly referenced and typically most plentiful substance is the psychoactive cannabinoid Δ9-tetrahydrocannabinol (Δ9-THC, also known simply as THC). Non-psychoactive cannabinoids, such as cannabidiol (CBD, a substance recently employed for use in the treatment of Dravet syndrome and Lennox-Gastaut syndrome), are also present in marijuana. Products colloquially referred to as "marijuana" are derived from the *Cannabis sativa* plant.

Cannabinoids bind CB receptors, of which there are two types (CB_1 located primarily in the brain, and CB_2 located in immune cells). CB receptors are found in differing concentrations throughout the body's organs and tissues. CB receptors have endogenous ligands, known as endocannabinoids (e.g., anandamide, and 2-arachidonoylglycerol [2-AG]), and together are known as the endocannabinoid system (ECS). Endocannabinoids are synthesized "on demand" and work as "retrograde messengers" and travel backwards to regulate the release of neurotransmitters; this results in decreased release of both inhibitory and excitatory neurotransmitters with the net effect functioning to regulate as a "rheostatic" regulator on neuronal excitability.

Δ9-THC is nearly completely metabolized by hepatic hydroxylation and oxidation (CYP2C9 and CYP3A4). The primary metabolite is 11-hydroxy-Δ9-tetrahydrocannabinol (11-OH-Δ9-THC), which is active and likely more potent than THC. 11-OH-Δ9-THC is subsequently metabolized via CYP2C9 to the inactive 11-nor-Δ9-THC carboxylic acid (THC-COOH). THC to 11-OH-THC conversion is generally higher when taken orally versus when smoked.

Synthetic cannabinoid receptor agonists (SCRAs; "K2," "Spice," etc.) also produce effects via interactions with CB_1 and CB_2 receptors, but have unique clinical effects[3] and are outside the scope of this guideline.

Dosing & Clinical Effects

Several dosage formulations exist for Cannabis products, but most can generally be categorized into 3 main delivery groups: products for inhalation, oral ingestion, and topical use. THC is 10-35% bioavailable when smoked, with effects occurring within minutes[2] with intoxication lasting up to 4 hours. Two to 3 mg of inhaled THC[4] may be intoxicating in naïve adult users. THC taken orally is 5-20% bioavailable with a peak

effect at 1 - 3 hours,[5] as little as 5 mg[4] may be intoxicating for a naïve adult user with effects lasting 12-24 hours.

Clinical manifestations vary based on age. In adults, Cannabis overall carries low risk for severe toxicity. Inebriating effects leading to poor judgment and concomitant use of other medications may lead to increased risk of falls or other injuries. In contrast, severe toxic effects of cannabis ingestions have been seen at doses at 5-300 mg in children,[4] and have been increasing,[6] particularly in U.S. states where decriminalization[7] and legalization[8] occur.[9,10] In *small children*, neurologic effects, including ataxia, hyperkinesis, lethargy, may occur; however, *coma and respiratory depression are the greatest concern*[11] and can occur even with small accidental ingestions.[12,13] Seizures may also occur.[14] Nausea and vomiting are also common.[14] Cardiovascular symptoms may include bradycardia and hypotension.[14] Severity of symptoms are dose-dependent, and more severe at lower doses in Cannabis-naïve children.[13] In a small series of 38 children from a pediatric emergency department, 3.2 mg/kg of THC led to observation and minimal interventions, 7.2 mg/kg led to inpatient admission and moderate interventions, and 13 mg/kg led to ICU admission and major interventions.[13]

In adults, acute intoxication is more varied than in children. Neurologic symptoms may include dysphoria, slurred speech, ataxia, agitation, and delirium. Psychosis may also occur, and may persist long after acute intoxication would be expected to resolve. Nausea and vomiting are common in acute intoxication, particularly with oral ingestions. In contrast to children, tachycardia and hypertension are more common in adults.[1] Cardiac complications such as dysrhythmias[15] and acute myocardial infarction[16] are associated with Cannabis use but are rare. Chronic, heavy marijuana use is associated with a clinical syndrome of abdominal discomfort, nausea, and cannabinoid hyperemesis syndrome (CHS).[17] CHS has become more common with the legalization and commercialization of Cannabis.[18,19]

Common dosing information for THC

- Starting doses of ingested THC for an adult is typically 1 - 10mg.
- Gummies/edibles are commonly available as 2 - 10 mg THC per piece, but can range as high as 60-80 mg per piece in some states.
    - In the state of Minnesota as of July 2022, THC edibles are limited to 5 mg per serving and 50 mg per package.[20]
- The average joint will contain 15-30 mg THC to start (depends on strain and concentration of flower product used; actual content subject to pyrolysis).

Clinical Effects of Cannabinoids other than Δ9-THC

CBD products – Cannabidiol or CBD products have gained much attention over the past several years, as their use and efficacy in refractory seizure disorders has been evaluated and found effective in clinical trials. The only commercially available CBD-only product that has been deemed to be "THC-free" is the prescription product "Epidiolex (which FDA approved for treatment of Dravet Syndrome and Lennox Gastaut syndrome).[21]

CBD is generally considered to be not psychoactive, however over-the-counter products sometimes contain impurities and other psychoactive compounds, including psychoactive cannabinoids.[22]

Δ8-THC products – Recently, there has been an increased availability of products claiming to contain the "less-potent" cannabinoid, Δ8-THC. Δ8-THC is chemically identical to Δ9-THC with the exception of a single carbon double-bond[23] (see figures).

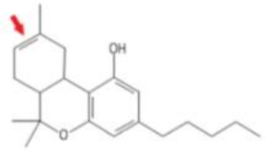

delta-8-THC

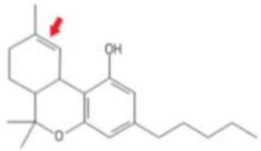

delta-9-THC

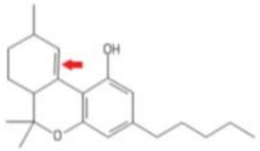

delta-10-THC

Δ8-THC binds cannabinoid receptors in a similar fashion to Δ9-THC, but are purported to have less potent effects on a mg-to-mg basis. To date, an exact potency comparison is not known, but it has been suggested online that Δ8-THC is approximately half as potent in overall effects including psychoactivity as those of Δ9 THC.[23] The safety of Δ8-THC in children, in particular, has a very poor evidence base.[24,25] Currently, Δ8-THC products are being sold through a purported legal loophole in the language of the 2018 "Farm Bill" (the Agriculture Improvement Act of 2018, which amends the definition of "Marihuana" is limited to extracts of Cannabis sativa "containing greater than 0.3 percent delta-9-tetrahydrocannabinol on a dry weight basis").[26] After passage of the 2018 Farm Bill, manufacturing of Δ8-THC by chemically modifying CBD became more common, and Δ8-THC products became more widely available. The legality of these products is currently being litigated.[27,28] Unlike CBD, users of Δ8-THC likely will have positive urine immunoassays for "THC" because of the similarity in chemical structures.[23] Recently, Δ10-THC products have been sold for similar purposes (see figure above); the potency and toxicity of Δ10-THC[29] is less certain than that of Δ8-THC.

Minnesota Poison Control System Treatment Guideline:

Cannabis (marijuana)

For Δ8-THC, Δ9-THC, and/or Δ10-THC, the following may be observed at home:

Children

- Lick, drop, or taste amounts & unwitnessed ingestions as long as patient is:
    - Asymptomatic
    - Parent/caregiver is available and responsible
    - Transport to hospital or access to 911 is readily available
    - See exceptions below regarding imminent expected sleep (e.g., naps, bedtime)
- Children treated with CBD oil, Epidiolex, or other CBD-only products for seizures who received an extra or increased dose of their product, and are asymptomatic.

Adults

- Most adult cases may be followed at home if the patient has mild to moderate symptoms such as somnolence, alterations in sense and time perception, depersonalization, and mood alterations.
    - Home monitoring in such patients is reasonable if the patient is able to drink fluids/maintain normal hydration

Recommendations for Home Care

- Monitor the patient (allowing for rest) in an area with minimal stimuli

Guidelines for Follow-up:

- Callers should be instructed to immediately call the poison center if the patient develops any cardiac symptoms (e.g. dyspnea, chest pain), any evidence of respiratory depression, or if neurologic status worsens.
- A follow-up call should be made 1 hour post-exposure in children, and 2-3 hours post-exposure in adults
    - Children monitored at home should receive a follow-up call once per hour until 3 hours post-exposure
        - Follow-up beyond 3 hours is at the discretion of the SPI
    - If the patient is asymptomatic *at 3 hours*, no further follow-up is required
    - If vomiting is present, cases should be followed until vomiting has stopped and the patient is able to drink fluids.

POISON Help

1-800-222-1222

Minnesota Poison Control System

Minnesota Poison Control System Treatment Guideline:

Cannabis (marijuana)

The following should be referred to a health care facility:

- Weight-based triage thresholds by age (for Δ8-THC, Δ9-THC, and/or Δ10-THC):[30]
  - 6 months - 5 years: > 0.2 mg/kg
  - 6 years - 12 years: > 0.3 mg/kg
  - 13 years - 79 years: > 0.4 mg/kg
- All children who are drowsy, regardless of dose ingested
- All children, regardless of dose ingested or symptoms, expected to sleep in the next 3 hours (i.e., those with exposures within 3 hours of their usual nap or bedtime).
- Patients with significant mental status changes, including confusion, obtundation or coma.
- Patients ingesting concentrated products meant for use by other delivery modes (e.g., oils, butters, shatters)
- Patients with persistent vomiting that are unable to drink fluids
- Patients deemed at risk for intentional-self harm (e.g., possible suicide attempts)

Treatment:

- Supportive care is the mainstay.
- A period of 3-4 hours of monitoring for asymptomatic children will typically facilitate safe discharge home.
- Treatments for acute vomiting in CHS:
  - The optimal antiemetics are likely antipsychotics.
    - Droperidol seems to be particularly effective.[31]
  - Some patients report relief with warm showers
  - Capsaicin cream (0.025% - 0.1%), applied to the abdomen, has been successfully used.[32,33] Some patients find this uncomfortable.
- Benzodiazepines are recommended for agitation, hallucinations, or seizures.

Initial date 8/2015; JC/SB 9/2020; BC/JC/CO 10/2022

Jon B Cole, MD, FACMT, FAACT
Medical Director

Carrie Oakland, PharmD, BCPS, CSPI
Clinical Supervisor

References:

POISON Help 1-800-222-1222 Minnesota Poison Control System

1. Welch SP, Smith TH, Malcolm R, Lichtman AH. Chapter 15: The Pharmacology of Cannabinoids. In: Herron AJ, Brennan T, eds. *The ASAM Essentials of Addiction Medicine, 3rd Edition.* Wolters Kluwer; 2020:78-83.

2. LaPoint JM. Cannabinoids. In: Nelson LS, Howland MA, Lewin NA, Smith SW, Goldfrank LR, Hoffman RS, eds. *Goldfrank's Toxicologic Emergencies, 11th Edition*. McGraw-Hill; 2019:1111-1123.

3. Tait RJ, Caldicott D, Mountain D, Hill SL, Lenton S. A systematic review of adverse events arising from the use of synthetic cannabinoids and their associated treatment. *Clin Toxicol* . 2016;54(1):1-13.

4. Wang GS. Cannabis (marijuana): Acute intoxication. Up to Date Online. Published April 21, 2022. Accessed October 8, 2022. https://www.uptodate.com/contents/cannabis-marijuana-acute-intoxication

5. Grotenhermen F. Pharmacokinetics and pharmacodynamics of cannabinoids. *Clin Pharmacokinet*. 2003;42(4):327-360.

6. Chartier C, Penouil F, Blanc-Brisset I, Pion C, Descatha A, Deguigne M. Pediatric cannabis poisonings in France: more and more frequent and severe. *Clin Toxicol* . 2021;59(4):326-333.

7. Wang GS, Roosevelt G, Le Lait MC, et al. Association of unintentional pediatric exposures with decriminalization of marijuana in the United States. *Ann Emerg Med*. 2014;63(6):684-689.

8. Wang GS, Le Lait MC, Deakyne SJ, Bronstein AC, Bajaj L, Roosevelt G. Unintentional Pediatric Exposures to Marijuana in Colorado, 2009-2015. *JAMA Pediatr*. 2016;170(9):e160971.

9. Heard K, Monte AA, Wang GS. Another Perspective on Cannabis and Emergency Medicine in Colorado. *West J Emerg Med*. 2019;20(6):855-856.

10. Roberts BA. Legalized Cannabis in Colorado Emergency Departments: A Cautionary Review of Negative Health and Safety Effects. *West J Emerg Med*. 2019;20(4):557-572.

11. Claudet I, Mouvier S, Labadie M, et al. Unintentional Cannabis Intoxication in Toddlers. *Pediatrics*. 2017;140(3). doi:10.1542/peds.2017-0017

12. Macnab A, Anderson E, Susak L. Ingestion of cannabis: a cause of coma in children. *Pediatr Emerg Care*. 1989;5(4):238-239.

13. Heizer JW, Borgelt LM, Bashqoy F, Wang GS, Reiter PD. Marijuana Misadventures in Children: Exploration of a Dose-Response Relationship and Summary of Clinical Effects and

Outcomes. *Pediatr Emerg Care*. 2018;34(7):457-462.

14. Stoner MJ, Dietrich A, Lam SHF, Wall JJ, Sulton C, Rose E. Marijuana use in children: An update focusing on pediatric tetrahydrocannabinol and cannabidiol use. *J Am Coll Emerg Physicians Open*. 2022;3(4):e12770.

15. Richards JR, Blohm E, Toles KA, Jarman AF, Ely DF, Elder JW. The association of cannabis use and cardiac dysrhythmias: a systematic review. *Clin Toxicol* . 2020;58(9):861-869.

16. Richards JR, Bing ML, Moulin AK, et al. Cannabis use and acute coronary syndrome. *Clin Toxicol* . 2019;57(10):831-841.

17. Razban M, Exadaktylos AK, Santa VD, Heymann EP. Cannabinoid hyperemesis syndrome and cannabis withdrawal syndrome: a review of the management of cannabis-related syndrome in the emergency department. *Int J Emerg Med*. 2022;15(1):45.

18. Myran DT, Pugliese M, Tanuseputro P, Cantor N, Rhodes E, Taljaard M. The association between recreational cannabis legalization, commercialization and cannabis-attributable emergency department visits in Ontario, Canada: an interrupted time-series analysis. *Addiction*. 2022;117(7):1952-1960.

19. Myran DT, Roberts R, Pugliese M, Taljaard M, Tanuseputro P, Pacula RL. Changes in Emergency Department Visits for Cannabis Hyperemesis Syndrome Following Recreational Cannabis Legalization and Subsequent Commercialization in Ontario, Canada. *JAMA Netw Open*. 2022;5(9):e2231937.

20. Legislature M. Minn. Stat. § 151.72 - SALE OF CERTAIN CANNABINOID PRODUCTS. Minnesota Legislature: Office of the Revisor of Statutes. Accessed October 8, 2022. https://www.revisor.mn.gov/laws/2022/0/98/laws.13.3.0#laws.13.3.0

21. FDA Approves First Drug Comprised of an Active Ingredient Derived from Marijuana to Treat Rare, Severe Forms of Epilepsy. U.S. Food and Drug Administration. Published June 25, 2018. Accessed October 12, 2022. https://www.fda.gov/news-events/press-announcements/fda-approves-first-drug-comprised-active-ingredient-derived-marijuana-treat-rare-severe-forms

22. Better Data for a Better Understanding of the Use and Safety Profile of Cannabidiol (CBD) Products. U.S. Food and Drug Administration. Published January 8, 2021. Accessed October 12, 2022. https://www.fda.gov/news-events/fda-voices/better-data-better-understanding-use-and-safety-profile-cannabidiol-cbd-products

23. Novak M. Delta-8-THC: The Ocho. Published online March 15, 2022.

24. Abrahamov A, Abrahamov A, Mechoulam R. An efficient new cannabinoid antiemetic in

pediatric oncology. *Life Sci*. 1995;56(23-24):2097-2102.

25. webPOISONCONTROL. #2198: delta-8 THC. webPOISONCONTROL. Accessed October 8, 2022. https://admin.webpoisoncontrol.org/mvc/algorithmspireport/#2198

26. Abernethy A. Hemp Production and the 2018 Farm Bill. U.S. Food and Drug Administration. Published July 24, 2019. Accessed October 8, 2022. https://www.fda.gov/news-events/congressional-testimony/hemp-production-and-2018-farm-bill-07252019

27. U.S. Drug Enforcement Agency. Implementation of the Agriculture Improvement Act of 2018. Federal Register: The Daily Journal of the United States Government. Published August 21, 2020. Accessed October 8, 2022. https://www.federalregister.gov/documents/2020/08/21/2020-17356/implementation-of-the-agriculture-improvement-act-of-2018

28. Herrington AJ. Federal Appeals Court Rules That Delta-8 THC Is Legal. Forbes. Published May 23, 2022. Accessed October 8, 2022. https://www.forbes.com/sites/ajherrington/2022/05/23/federal-appeals-court-rules-that-delta-8-thc-is-legal/?sh=bbce99a57d8a

29. webPOISONCONTROL. #2363: delta-10 THC. webPOISONCONTROL. Accessed October 8, 2022. https://admin.webpoisoncontrol.org/mvc/algorithmspireport/#2363

30. webPOISONCONTROL. #1053: tetra-hydrocannabinol (THC). webPOISONCONTROL. Accessed October 8, 2022. https://admin.webpoisoncontrol.org/mvc/algorithmspireport/#1053

31. Lee C, Greene SL, Wong A. The utility of droperidol in the treatment of cannabinoid hyperemesis syndrome. *Clin Toxicol* . 2019;57(9):773-777.

32. Graham J, Barberio M, Wang GS. Capsaicin Cream for Treatment of Cannabinoid Hyperemesis Syndrome in Adolescents: A Case Series. *Pediatrics*. 2017;140(6). doi:10.1542/peds.2016-3795

33. Dean DJ, Sabagha N, Rose K, et al. A Pilot Trial of Topical Capsaicin Cream for Treatment of Cannabinoid Hyperemesis Syndrome. *Acad Emerg Med*. 2020;27(11):1166-1172.
